# Hemorrhage in Typhoid Fever

**Published:** 1911-06

**Authors:** C. W. Strickler

**Affiliations:** Atlanta, Ga.


### HEMORRHAGE IN TYPHOID FEVER.

By Dr. C. W. Strickler, Atlanta, Ga.

Hemorrhages from the bowel in typhoid fever is one of the most serious complications we have to deal with, and on account of this the individual study of such accidents is not only interesting but imperative if we hope to handle these cases successfully.

Even in the light of our present knowledge and clinical experience we feel almost helpless in the face of this emergency. Realizing this, I have made as careful an individual study of each case that has come under my observation as possible, hoping that I might learn how better to help these unfortunates. My experience has cost me something which has seemed to be of great value. I will, therefore, very briefly give you the results of my observations.

In my study of these cases I have met with a certain type of patient that almost invariably bleeds so frequently, indeed, that I have prognosticated hemorrhages, and although I have used the preventive remedies recommended by the best authorities, the prognosis has come true.

It is somewhat difficult to describe this type of patient. They resemble very much the tubercular patient or certain types of neurasthenics without the extreme nervousness of the latter. The skin is usually very fair, thin, the cheeks are rosy, the hair is very fine, the eye-lashes are long, the eyes are bright. The pulse is apparently full and strong, but really very compressible: the arterial tension being actually low. The pulse becomes dicrotic early. I have noted the fact that these cases almost in every instance were more or less alarmed over their illness.

There seem to be three different varieties of hemorrhages:

1st. The patient has a copious, bloody, fluid discharge, that continues in spite of treatment and terminates fatally in a few minutes or hours. Collapse is grave from the beginning, and the utter futility of attempting any kind of assistance is evident.

2nd. There seems to be another variety in which the hemorrhage is severe enough to cause collapse but not so severe as in the above. The blood is passed; some fluid and some clotted, and under proper treatment a certain number of these cases recover.

3rd. There seems to be another variety still in which we have frequent but small hemorrhages. Usually clotted when passed. The pulse in these cases is little if at all affected. The temperature is either not affected at all or gradually comes down to normal and the patient rapidly recovers. One such case had apparently 24 different hemorrhages but recovered. Clinically this has been the course of the cases that I have personally treated, and had the pleasure of observing in the hands of my colleagues and friends. From my study of these cases I formed some conclusions in regard to treatment, which are as follows:

In Class 1, I have found all efforts fruitless, and am of the opinion that all such cases are hopeless from the beginning. Yet the same treatment outlined in Class 2 may be used.

In Class 2, it is important to stop all previous treatment and absolutely stop the intestinal movements by morphine given hypodermically. No food or liquid should be given by the mouth until several days have elapsed, unless the patient's condition positively demands our taking this risk. Necessary fluids should be given very slowly by the bowel. When feeding is started the nutriments should be not too fluid in order to keep down intravascular tension.

When you are sure you are dealing with the third class, liquid nourishments may and must be kept up, and seem to do no harm. If paristalsis is too vigorous, small doses of morphine are indicated.

There are certain remedies and means that have been used some for the control of the hemorrhage and others to save the life of the patient. Some of these are apparently of some value, others are theoretically wrong and clinically have seemed to hasten the end. In the first class I will mention chloride and lactate of calcium, as probably useful agents to prevent and control some hemorrhages. In hemorrhagic typhoid, fortunately very rare, these remedies, I believe, would be of some value. Turpentine is sanctioned by long usage and fathered by many able practitioners, as especally useful in cases in which the hemorrhage is small. The lead and opium pill is still used and recommended by many. It has always seemed to me doubtful if adrenalin chloride and normal salt solution are ever proper agents to use, either to control hemorrhage or to save life. If we get the physiological effect of adrenalin we get the opposite conditions to that which is desired, viz.: increased arterial tension. If it has any local effect upon the bleeding point, I do not know it. In the cases in which I have used it, it has seemed to do no good. Even if it could be locally applied, I doubt whether any good would come from it, even though applied to the bleeding point.

While normal salt solution has been used for a long time by many and able practitioners, I don't believe it is of any real value but productive of harm. In spite of the statement made in some text books that it favors clotting I have never in a single instance seen any good coming from its use, but more than once a fatal hemorrhage has apparently been started. It is a mechanical means, and although used to save life, brings about the very condition that defeats our purpose.

I believe the salvation of these cases lies in the fact that they have nearly but not entirely bled to death. It certainly is reasonable to believe that such a case stands a much better chance by keeping the tension of the vessels as low as possible, in order that clotting and organization of the clot may be successfully accomplished.

In all cases of typhoid had the coagulability of the blood estimated early in the disease, so that if below par proper steps could be taken to increase it, hemorrhages in these cases would probably be of less frequent occurrence.

Candler, Bldg.. Atlanta, Ga.

